# Studies on Numerical Buckling Analysis of Cellulose Microfibrils Reinforced Polymer Composites

**DOI:** 10.3390/ma16030894

**Published:** 2023-01-17

**Authors:** Venkatachalam Gopalan, Mugatha Surya Vardhan, Vishal Thakur, Annamalai Krishnamoorthy, Vignesh Pragasam, Mallikarjuna Reddy Degalahal, Pitchumani Shenbaga Velu, A. Raja Annamalai, Chun-Ping Jen

**Affiliations:** 1 Centre for Innovation and Product Development, Vellore Institute of Technology, Chennai 600127, India; 2 School of Mechanical Engineering, Vellore Institute of Technology, Chennai 600127, India; 3 Associate Project Engineer, EinNel Technologies, Chennai 600073, India; 4 School of Mechanical Engineering, Vellore Institute of Technology, Vellore 632014, India; 5 Centre for Innovative Manufacturing Research, Vellore Institute of Technology, Vellore 632014, India; 6 School of Dentistry, College of Dental Medicine, Kaohsiung Medical University, Kaohsiung 80708, Taiwan; 7 Department of Mechanical Engineering and Advanced Institute of Manufacturing for High-Tech Innovations, National Chung Cheng University, Chia-Yi 62102, Taiwan

**Keywords:** finite element model, microfibrils, buckling analysis, ANOVA, regression analysis

## Abstract

Scientists are drawn to the new green composites because they may demonstrate qualities that are comparable to those of composites made of synthetic fibers due to concerns about environmental contamination. In this work, the potential for using the produced green composite in different buckling load-bearing structural applications is explored. The work on composite buckling characteristics is vital because one needs to know the composite’s structural stability since buckling leads to structural instability. The buckling properties of composite specimens with epoxy as the matrix and chemically treated cellulose microfibrils as reinforcements are examined numerically in this study when exposed to axial compressive stress. The numerical model is first created based on the finite element method model. Its validity is checked using ANSYS software by contrasting the critical buckling loads determined through research for three samples. The numerical findings acquired using the finite element method are then contrasted with those produced using the regression equation derived from the ANOVA. The utilization of the created green composite in different buckling load-bearing structural applications is investigated in this study. As a result of the green composite’s unaltered buckling properties compared to synthetic composites, it has the potential to replace numerous synthetic composites, improving environmental sustainability.

## 1. Introduction

Polymer composites are used in many structural applications. Thus, it is crucial to promote green composites comprised of natural fibers to conserve the environment for future generations. The green composite produces fewer carbon emissions during destruction than a synthetic composite since green ingredients were employed to make it. As a result, there is less environmental contamination and a more stable, green ecosystem. Due to the use of natural fiber composites in load-bearing applications, including automotive, construction and others, researchers have been studying natural fiber composites under static and dynamic loads [1]. Due to the structural instability of composite structures, the structure will attain a failure in various ways, such as fatigue failure and buckling failure. Due to the axial compression pressure or heat load, composite constructions are frequently susceptible to buckling failure [2]. The natural fiber polymer composite constructions may experience an axial compression load throughout their service since they are employed in a variety of load-bearing applications [3]. A natural fiber-reinforced polymer composite needs to be able to bear stresses and maintain stability throughout its full life cycle. Large compressive stresses, however, might be applied to the structure, leading to a buckling failure. Buckling-related failures of natural fiber-reinforced polymer composite structures occur at loads many times below the material’s yield strength [4]. It is commonly known that a structure’s buckling is influenced by its slenderness ratio. Several studies have been conducted to investigate the mechanical properties and other physical parameters of natural fiber-reinforced polymer composites [5]. It is crucial to analyze the structural performance of novel materials throughout service in addition to their characterization. Hence it is important to examine the buckling behavior of natural fiber-reinforced polymer composite structures to enhance their usage in various applications [6].

The buckling properties of laminated composite plates were studied utilizing a plate with internal holes, shear deformation, local effects, nonlinear stress-strain behavior, sandwich construction with other materials, hygro-thermal effects, external stiffeners, post-buckling behavior and early faults [7]. They identified the improvements during buckling loads of a rectangular plate. Additionally, the effects of laminated composite plates with different boundary conditions, thickness, aspect ratios and membrane stiffness on the buckling loads were studied [8]. The modified rectangular plate specimen was subjected to buckling load to evaluate the effectiveness of composite plates subjected to bi-axial stress [9]. For numerical simulations, shell elements such as Shell181 and Shell281 are suitable to model rectangular plates. The plate/shell concept theory reduces the plate issue to a surface model with thickness-dependent strain and deformation suppositions for shell components [10]. The buckling performance of a thin-walled carbon/epoxy laminated circular cylindrical composite shell under combined axial and torsional loading is evaluated both analytically and experimentally. It was found that the stiffness eccentricity played a major role in the amount of axial buckling load than that of the combined load [11]. A laminated composite cylinder with cut-outs was subjected to buckling and post-buckling examination. The combined effect of internal pressure and compression was examined. The results showed that the buckling load of a compression-loaded cylinder was highly influenced by internal pressure, cut-out and orientation. Thus, buckling analysis is essential for detecting localized delamination failure or structural instability in plate structures [12]. Natural fiber-reinforced composite beams’ buckling and free vibration behaviors were experimentally subjected to axial compression. A numerical study using the finite element technique was used to validate a critical buckling load that was calculated experimentally. It was shown that the number of layers increased the composite laminate’s buckling strength. It was also discovered that the critical buckling load was impacted by the weaving pattern of a woven fabric, with the basket-type weaving model providing a higher buckling strength [13]. To explore the buckling and vibration properties of a partly or internally cracked natural fiber-reinforced composite plate with corner point supports, a new symplectic analytical methodology integrated with the finite element method has been developed. According to the authors, a plate with an interior fracture would reduce the critical buckling load and the natural frequency more than a plate with a surface crack [14]. Various parametric studies were also performed using the proposed finite element model by considering the different parameters such as the ply orientation, the aspect ratio and the number of layers. The elastic constants were determined for the numerical simulation using a new theoretical approach, especially for the natural fiber-reinforced polymer laminated composites [14].

Plant fibers are comprised of cellulose, lignin and hemicellulose, with numerous microtubules aligned along the fiber. Fiber stiffness and strength were directly related to the primary constituent of plant fibers, cellulose. Response Surface Methodology was used to set up different combinations by considering different factors. Numerical and experimental studies were also performed on the dynamic properties of uniform plant fiber-reinforced polymer laminated composite plates, where the elastic constants of the composite lamina were used [15]. The alkali treatment method is one of the simplest, most cost-effective procedures for improving the bonding of natural fibers to epoxy resin. Several researchers looked at the effect of the coupling agent sodium hydroxide in alkali treatment. The findings demonstrated that varying concentrations of sodium hydroxide have distinct effects on fiber surface morphology [16,17,18]. Sodium hydroxide was used as an alkali treatment because it converts fibers’ smooth surface to a rough surface by making undulation on the surface. It indicated that the matrix in the cell wall was removed after alkali treatment. The author reported that the pore sizes and void ratios vary depending on cellulose origin and as well as treatment history. These pores are important for dissolution because it provides spaces for the diffusion of solvent chemicals into fibers. Na-Cellulose-I lattices were formed when excess sodium hydroxide was removed. Cellulose-II, having a more stable lattice in comparison to Cellulose-I, was formed by rinsing the cellulose with water and removing the sodium ions from the cellulose [19,20,21,22]. The fiber is affected by the alkaline treatment in two ways. As a result, mechanical interlocking was improved, and the surface roughness and cellulose exposure to reaction sites were both increased. It was observed that mechanical interlocking has a long-term influence on fiber mechanical behavior, notably the strength and stiffness of fibers [23,24,25].

Kabir et al. [26] studied the surface treatments of natural fibers to maximize the composites’ bonding strength and stress transferability. The overall mechanical properties of natural fiber-reinforced polymer composites were highly dependent on the morphology, aspect ratio, hydrophilic tendency and dimensional stability of the fibers. Harish et al. [27] evaluated the mechanical properties of coir-reinforced composites. The tensile results showed the suitability of coir fiber for low load-bearing applications. Rashed et al. [28] studied the tensile strength of jute fiber-reinforced composites. The effects of parameters, such as alkali treatment (compared to no treatment), fiber size (1, 2 and 4 mm) and fiber loading (5, 10 and 15 wt%) on the tensile strength were considered. They analyzed the tensile behavior and performed fractographic observations. Akil et al. [29] reviewed kenaf fiber-reinforced composites. Kenaf fibers are readily available and are used as reinforcement in various ways. NaOH-treated kenaf fiber increases the tensile and flexural properties of epoxy composites, but the thermal resistance decreases compared to untreated kenaf fiber-reinforced composites. Senthilkumar et al. [30] evaluated the mechanical and vibrational properties of pineapple leaf fiber-reinforced composites. They prepared the PALF polyester composites by the hand lay-up process and then compressed the materials using a compression testing machine. They analyzed the tensile, flexural and vibration results and found that an increase in PALF reinforcement increases the mechanical strength and reduces the damping ratio of the composites. They suggested that 45 wt% PALF composites are better suited for structural applications. 

Ozbek [31] investigated the effect of silica nanoparticles (NS) on the buckling characteristics of Kevlar/epoxy fiber-reinforced composite laminates. The results revealed that decreases in the length of samples resulted in significant increases in axial and lateral buckling characteristics. Rozylo et al. [32] studied the failure phenomenon of carbon fiber-reinforced plastic columns with three distinct lay-up patterns of the composite. It was observed that the dominant failure form occurred in the region representing the end sections of the composite structures (short 180 mm columns). Moreover, the delamination phenomenon usually occurs just before structural failure. Debski et al. [33] investigated the effect of eccentric compressive load on the stability, critical states and load-carrying capacity of thin-walled composite Z-profiles. Compressive fiber damage began in Plies 2 and 7 in the squeezed composite structure. The damage process evolved into a complex failure mechanism, causing the structure to lose its load-carrying capability when all mixed damage initiation requirements were met in the damaged area.

**Figure 1:**
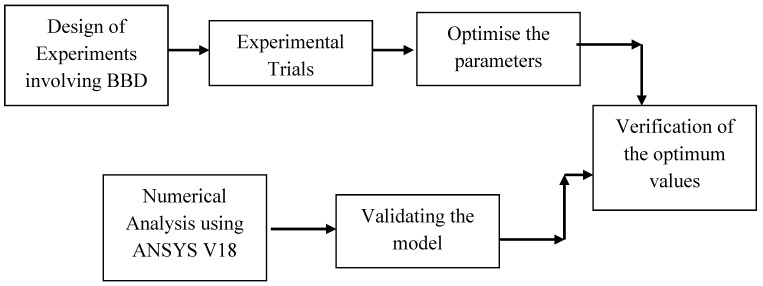
Research Flowchart.

Although many researchers studied the mechanical and flexural characteristics of different fiber/polymer combinations, they still have limited knowledge related to the buckling behavior of composites reinforced with natural cellulose microfibrils. However, they have not carried out optimization methods such as the response surface approach to determine the ideal critical buckling load for natural fiber composites. In the present study, the critical buckling stress of a composite reinforced with cellulose microfibrils is analyzed using Box--Behnken response surface design, and the desired parameter result is optimized. The research flowchart is mentioned in Figure 1. The impact of three different parameters, including fiber volume% (*w*/*w*), fiber diameter (µm), and sodium hydroxide % (*w*/*w*) on buckling behavior (dependent variable), is investigated.

## 2. Methodology 

Raw banana stem fibers were processed to micron size, acquired from local vendors (ECO Green unit, Coimbatore, TN, India), and used as a filler. Chemicals such as NaOH, HCl and demineralized water were used to remove cellulose microfibrils (SRL Chemicals, Sigma Aldrich, and NICE Chemicals, Chennai, TN, India). Epoxy LY556 and hardener HY951 were purchased from S.M. Composites, Chennai, TN, India.

**Figure 2:**
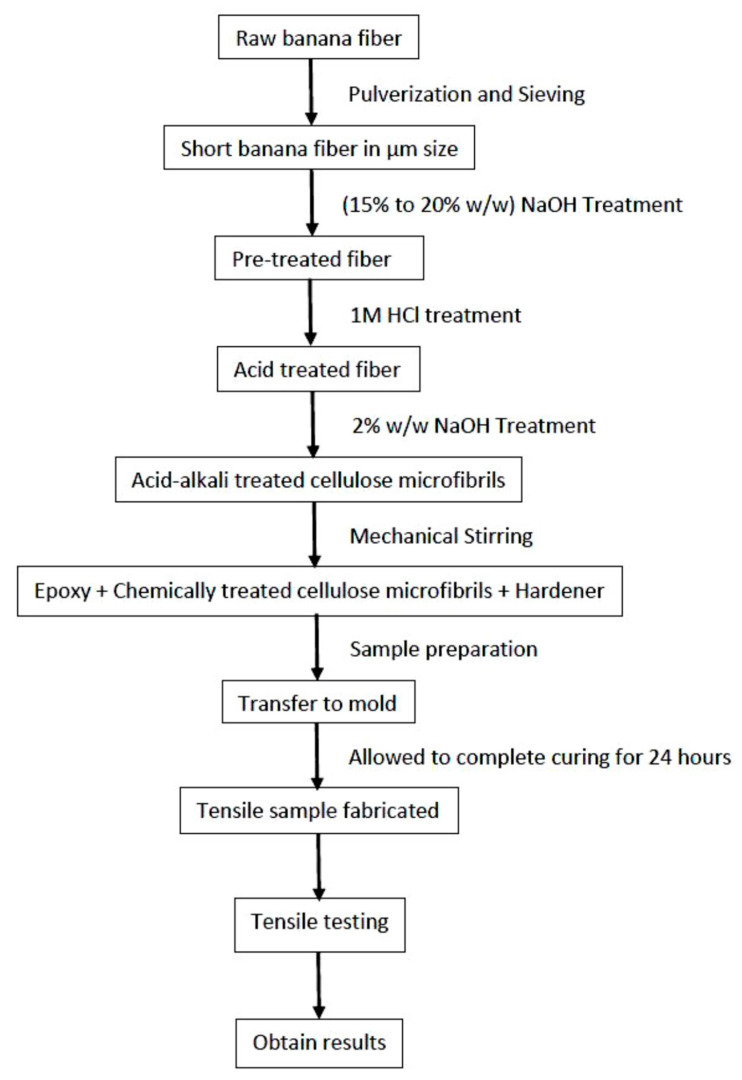
Process Flowchart for Tensile sample preparation [34].

Raw banana fibers were cut to 4–5 mm lengths and prewashed with demineralized water to remove dirt and impurities. Then, the fibers were air-dried for two days to remove excess moisture. Fully dried banana fibers were powdered by the pulverizing process (Saral Pulverizer, Gujarat, India) and size separation was performed using a sieve shaker. Figure 2 describes the procedure for the chemical treatment of powdered banana fiber to prepare cellulose microfibrils for reinforcement of epoxy composites. The chopped banana fibers were pretreated with different *w*/*w* percentages of sodium hydroxide (NaOH) solution for 2 h. Then, the fibers were washed several times with distilled water. The pretreated banana fibers were hydrolyzed using a 1M HCl solution at 80 °C ± 5 °C for 2 h. Then, the fibers were washed several times with demineralized water. The acid-hydrolyzed fibers were treated again with a 2% (*w*/*w*) NaOH solution for 2 h at 60 °C ± 5 °C. The acid-alkali-treated fibers were washed several times with demineralized water until the pH reached 7. These acid-alkali-treated fibers had more cellulose microfibrils and less pectin, hemicelluloses and lignin.

The Euler critical formula is utilized for predicting the buckling load of the supporting plate with different boundary parameters. Euler’s Critical Load Formula for Plate is presented in Equations (1) and (2).

D = E h ^3^/12(1 − ν^2^) (1)

N_xcr_ = k_c_ π^2^ D/b^2^ (2)

where E represents Young’s modulus of Text plates, ν represents Poisson’s ratio, k_c_ = a/b Buckling Coefficient, h- thickness, D- Flexural rigidity of the plate per unit length and N_xcr_-critical buckling load. The plate thickness, width, Young’s modulus and Poisson’s ratio are a = 100 mm, E = 5249.27 MPa, v = 0.34, h = 0.75 mm and a/b = 1, respectively. One can theoretically calculate the critical buckling load using this Euler critical formula and these property values. The critical value factor in this expression represents the Euler load for a strip of unit width and length ‘a’. The second factor, ‘b’, denotes the proportion of greater stability gained by the continuous plate compared with that of an isolated strip. Table 1 shows that the epoxy reinforced with 20% NaOH-treated filler (run no. 14) exhibits higher tensile stress and modulus compared to the other samples.

### Response Surface Methodology

In the study, a sophisticated statistically verified prediction model is used to conduct and evaluate trials to determine the optimal combination and the impact of various elements, such as a change in sodium hydroxide %, a change in fiber diameter and a change in volume percentage, on the buckling characteristics. The experimental design in this work employs the Response Surface Methodology’s Box--Behnken design. Table 2 lists the process parameters and the three levels at which each parameter is evaluated. Box--Behnken design is used to generate higher-order response surfaces using fewer required runs than a standard factorial technique [34]. The design uses the twelve middle edge nodes and three center nodes to fit a 2nd order equation.

In MINITAB software, these levels and factors are utilized to frame the different combinations and represent the results, as shown in Table 3. 

## 3. Results and Discussion

### 3.1. Buckling Analysis

**Figure 3:**
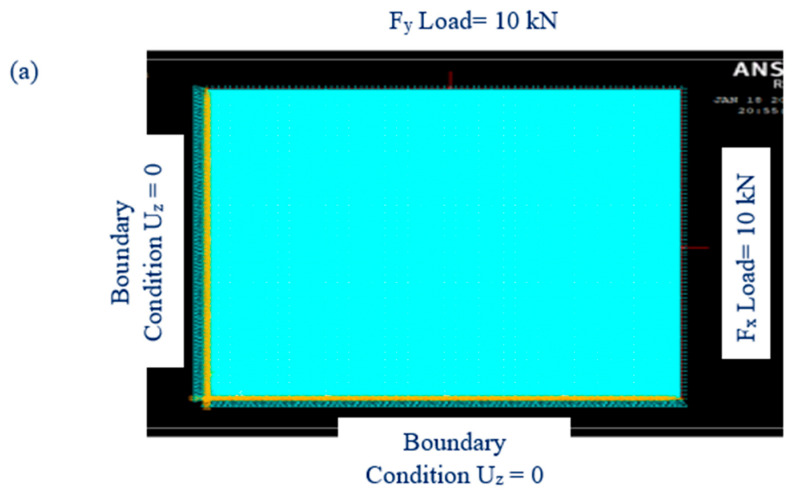

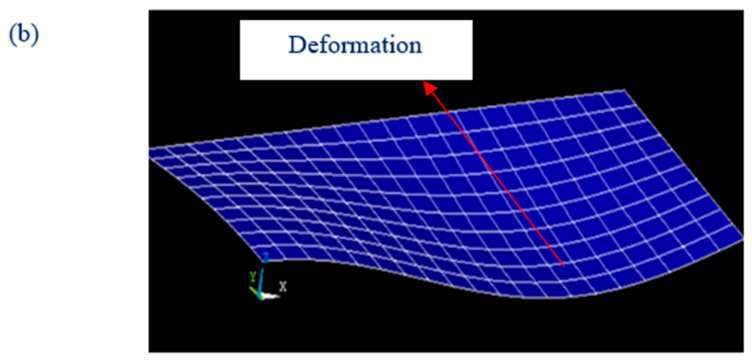
(**a**) Meshed model (**b**) Buckled model under simply supported boundary condition.

This examination is performed to find out how well laminated composite samples buckle under axial compressive stresses. Software based on the finite element method, ANSYS V.18, is used to do the numerical analysis. The critical buckling loads and load factors of 15 composite samples are determined. Shell 181, a four-node element with six degrees of freedom at each node, was utilized to simulate the laminated composite in this investigation. The lay-up technique is applied to define the orientation of the sample and the number of layers under sections. An evenly distributed 10 kN compression load is applied. An axial compression load is applied to a mesh-supported boundary condition. The plate is supported with loaded edges, and all the loaded edges are simply supported. Uz = 0, as shown in Figure 3a. Simply supported end conditions are used, as shown in Figure 3b. The critical buckling load is determined by multiplying the load factor by the applied load [35]. It reveals the buckling factor found numerically using ANSYS 18; the results are mentioned in Table 4. The deformation was applied in the longitudinal direction to the upper & side parts of the model, representing the upper grip. All the other degrees of freedom were fixed for this part. The displacement was a linear function up to a chosen maximum deformation limit. The essential output of this model is the critical buckling force, i.e., the force for the onset of buckling. The result of the buckling deformation is shown in Figure 3b. The model allows the whole bottom grip of the specimen to move freely in the X-Y plane. This simplification is expected to give lower buckling force values in the model than in the experiments. In general, it is observed that the calculated values from the model are smaller than the measured values from the experiments.

### 3.2. Numerical Analysis

All 15 samples are numerically simulated and mentioned in Table 5. ANSYS 18 is utilized to calculate the critical buckling loads for each of the 15 samples. An analysis of variance in MINITAB software is carried out using data from the numerical simulation ANSYS 18. Maximum critical buckling loads are discovered in the samples with a sodium hydroxide ratio of 18.03% *w*/*w*, a fiber diameter of 333.3 µm, and a fiber volume of 4.14% *w*/*w* based on numerical simulation findings. 

### 3.3. Comparison of Numerical and Theoretical Results

In order to validate, the developed numerical model is compared with the theoretical model available in the literature [34]. The finite element method/developed numerical model is validated by comparing numerical and theoretical critical buckling loads for three samples using ANSYS V.18. The validation indicates a positive sign as the error percentage is less than two. The error percentage of a random three from 15 samples is listed in Table 6.

### 3.4. Analysis of Variance Assessment for the Regression Model and Regression Equation

Utilizing Minitab software, the Box--Behnken design of the Response Surface Methodology approach is used to investigate the major impact of three variables: sodium hydroxide percent (*w*/*w*), fiber diameter and fiber volume on the critical buckling load of composite plates. The quadratic polynomial regression equation for the critical buckling load is derived from the fitting curve, as shown in Equation (3).

A = sodium hydroxide ratio (percentage) (*w*/*w*), B= fiber diameter (µm), C = fiber volume % (*w*/*w*)

Critical buckling load = (−12628 + 1320 A + 3.36 B + 734 C − 35.64 A*A − 0.00426 B*B − 82.6 C*C − 0.050 A*B − 4.35 A*C + 0.091 B*C)(3)

### 3.5. Comparing the Critical Buckling Load of the Finite Element Analysis and the Regression Equation

The resulting regression equation is then used to compute the critical buckling load for each combination. Table 7 details the results of this comparison with those obtained using finite element analysis software.

**Figure 4:**
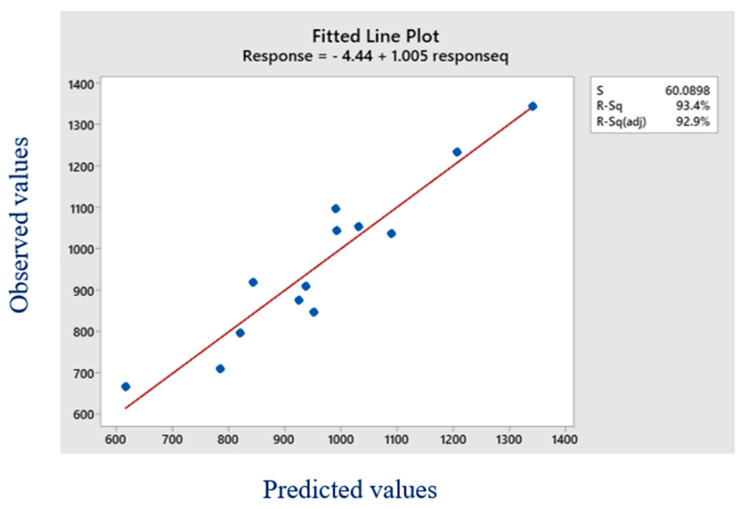
Graph between the predicted value and actual value.

Table 7 shows that, except for a few circumstances, the average variation is between 3–5 and less than 11 percent. The comparison proves the validity of the created regression model and the derived regression equation. This demonstrates the stability of the finite element method model, and a similar model is utilized for analyses of the same type, as shown in Figure 4. The critical buckling load for each possible combination may also be calculated using this regression equation. The ANOVA is also carried out for the critical buckling load quadratic model. The *p*-value for the model is <0.02, demonstrating its importance. The modified R^2^ value of 0.82 is quite similar to the projected R^2^ value of 0.93. The model is noteworthy since the gap between the anticipated R^2^ value and the adjusted R^2^ value is likewise smaller than 0.11. The model summary values are shown in Table 8. Figure 4 presents the predicted vs. actual values of R^2^. The points in the graph are a bit scattered, which indicates that the predicted and actual values are a bit wider, but the points are still within a range.

**Figure 5:**
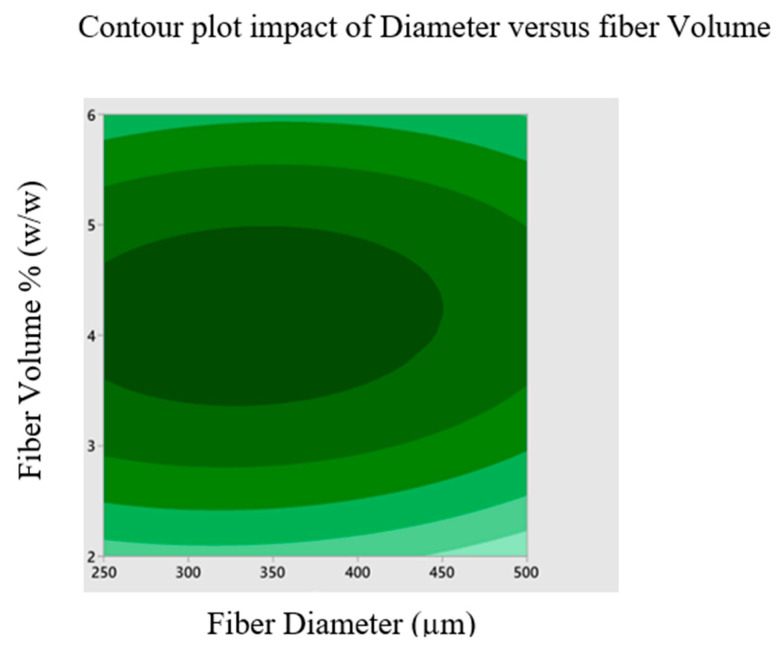
Contour plot impact of diameter versus fiber volume.

Figure 5 depicts the contour plot exhibiting the influence of fiber diameter and volume on buckling load while maintaining a sodium hydroxide concentration of 17.5 percent *w*/*w*. According to the graph, the maximum buckling load is attained at a fiber volume of 4% *w*/*w* ratio and a fiber diameter of 375 μm. It is found that the fiber volume should be a 4% *w*/*w* ratio to have the greatest critical buckling load and that the fiber diameter does not affect the tensile critical buckling load, shown in Figure 5.

### 3.6. Effect of Parameter on Critical Buckling Load

**Figure 6:**
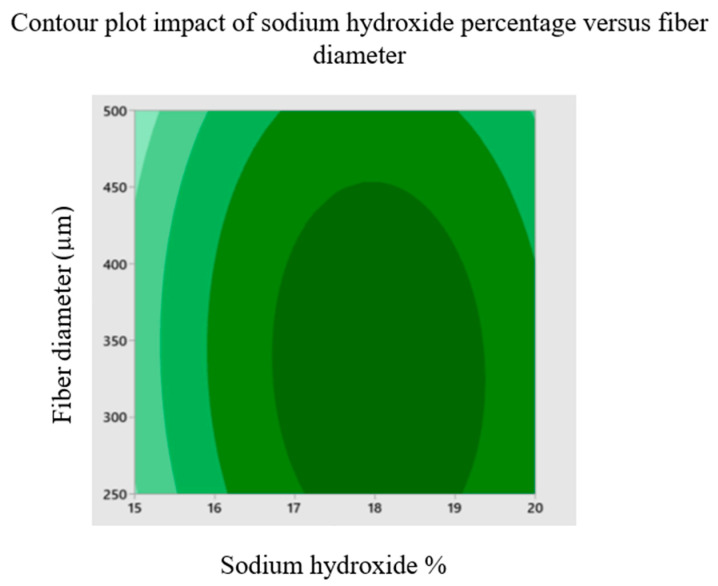
Contour plot impact of sodium hydroxide percentage versus fiber diameter.

**Figure 7:**
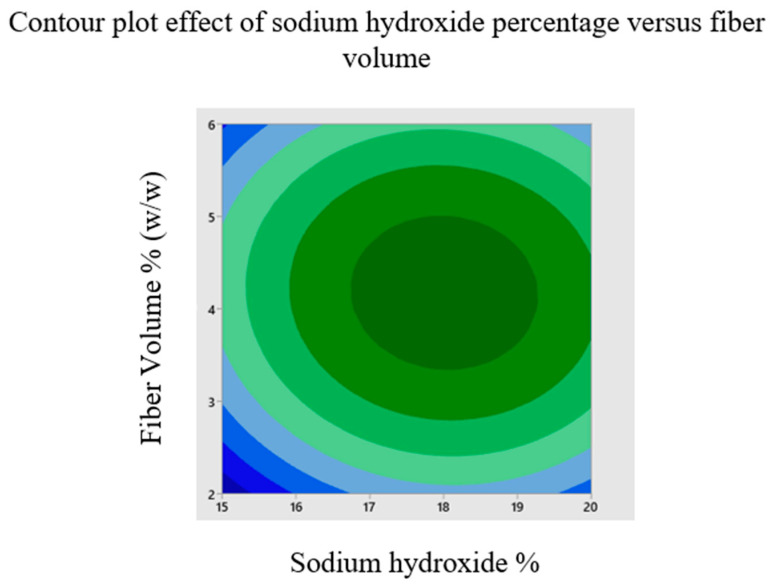
Contour plot effect of sodium hydroxide percentage versus fiber volume.

The effects of sodium hydroxide percentage, fiber size and fiber volume on the buckling load of chemically treated cellulose microfibril-reinforced epoxy composites are investigated using a three levels/factors Box--Behnken design model. Contour plots are created using the model to display the primary and interactive impacts between the response and independent variables. To better comprehend the interactions between the variables and to identify the ideal conditions, these graphs are created between two independent variables while keeping one variable constant. Figure 3 depicts the contour plot influence of fiber diameter and volume on buckling load while maintaining a sodium hydroxide percentage of 17.5 percent *w*/*w*. According to the graph, the maximum buckling load is attained at a fiber volume of 4% *w*/*w* ratio and a fiber diameter of 375 μm. Figure 6 depicts the contour response graph indicating sodium hydroxide percentage and fiber diameter influence buckling load when fiber volume is maintained at 4% *w*/*w*. Maximum buckling load is attained at 18.5% sodium hydroxide percentage by weight. Figure 7 depicts the contour response plot of the sodium hydroxide percentage and fiber volume influence on the buckling load with a fiber diameter of 375 μm. Figure 7 shows that the maximum buckling load is reached at a sodium hydroxide percentage of 18.5% and a fiber volume of approximately 4%. Surface plot effects demonstrate that the natural surface area of fiber grows as the sodium hydroxide percentage increases. This increase in the surface area helps the matrix to wet the fiber effectively and creates an excellent connection with the natural fiber [36], as shown in Figure 6. Additionally, chemical processing enhances the possibility of exposing more cellulose fibrils to the matrix material, increasing the mechanical strength of composites reinforced with cellulose fibrils, as shown in Figure 7. NaOH treatment is a process of alkalization on natural fibers, which may improve the bonding. It is indicated that the best chemical treatment is modifying the surface of natural fibers offering better adhesion with resin polymer composites. In addition, the mechanical properties were observed to enhance with the increasing NaOH percentage [37].

### 3.7. Verification and Optimization of the Model

Derringer’s desirability function optimization process is used to obtain the optimal parameters to produce the largest critical buckling load. It depicts the desirability ramp for maximizing the input variables to provide the optimum results, as shown in Figure 7. In order to obtain the best results for the maximum critical buckling load of 1361.61 N, it is recommended to modify the input variables of fiber diameter to 333.3 m, sodium hydroxide ratio to 18.03% *w*/*w*, and fiber volume to 4.14% *w*/*w* ratio, as shown in Table 9 and Table 10. 

### 3.8. Verification of Optimum Value

Table 11 and Table 12 show the critical buckling load calculated with a numerical model and the percentage departure from the optimal condition to validate the optimization result. The deviation between the model and the experiments is 1% between the average of experiments and the model. The model shows a significantly better correlation for the analysis. In general, it is observed that the calculated values from the model are smaller than the measured values from the experiments. The verification of optimization solutions is conducted via the finite element method. The buckling factor is 135.11, and the buckling load is 1351.1 N. The critical buckling load was significant depending on the thickness of the plate. As is evident, the critical buckling load was significantly larger with an increase in the thickness of the plate [38]. Table 13 shows the variation of the buckling factor depending on the sodium hydroxide percentage and fiber volume percentage of the reinforced composite. The buckling factor is high at higher concentrations of the sodium hydroxide and fiber volume percentage.

## 4. Conclusions

The Box--Behnken design approach is used in the present work to achieve optimum process parameters for the critical buckling load of polymer composite. Using a three-level/parameter model, the variables sodium hydroxide percent (*w*/*w*), fiber diameter (μm) and fiber volume percent (*w*/*w*) are taken into account. Buckling load results are tabulated and analyzed as well as optimized with Minitab software. The analytical results and the expected results show excellent agreement. The contour diagram provides a clear explanation of how the factors affect the critical buckling load. Its optimized value is 1361.64 N at the input variables of 18.30% *w*/*w* ratio of sodium hydroxide, 333.3 µm of fiber diameter and 4.14% *w*/*w* of fiber volume. This work demonstrates how well Box--Behnken design can be used to model cellulose microfibril-reinforced polymer composites and achieve optimal outcomes quickly and with a small number of experimental runs.

## Figures and Tables

**Table 1:** Experimental and predicted values of tensile stress and tensile modulus of composites [34].

Run No	Tensile Stress (MPa)			Young’s Modulus (MPa)		
	Experimental	Predicted	Error %	Experimental	Predicted	Error %
1	33.68	34.81	−3.35	6848.70	7168.77	−4.67
2	26.12	28.77	−10.14	5249.27	4709.72	10.27
3	36.26	35.98	0.76	10,527.24	10,309.23	2.07
4	36.59	36.60	0.02	8176.21	7804.89	4.54
5	35.80	35.37	1.12	8169.82	9307.41	−13.92
6	25.42	25.77	−1.39	6239.62	7214.06	−15.62
7	28.71	28.61	0.35	8510.55	8170.59	4.03
8	36.26	35.98	0.76	10,527.24	10,309.23	2.07
9	40.78	39.73	5.03	6618.06	7214.06	−9.21
10	35.55	33.04	7.06	8311.05	8170.59	1.69
11	25.37	26.84	−5.75	5505.91	5666.25	−2.92
12	37.69	36.74	2.53	7243.13	6212.24	14.23
13	36.26	35.98	0.76	10,527.24	10,309.23	2.07
14	41.48	44.57	−7.45	9734.19	9307.41	4.38
15	28.69	27.40	4.53	7054.35	7804.89	−10.64

**Table 2:** Factors and levels.

Factor	Variables	Coded Variable Levels		
		−1	0	+1
X_3_	Fiber volume% (*w*/*w*)	2	4	6
X_2_	Fiber diameter (µm)	250	375	500
X_1_	NaOH% (*w*/*w*)	15	17.5	20

**Table 3:** Response Surface Methodology.

Run No.	Fiber Volume% (*w*/*w*)	Fiber Diameter (µm)	NaOH% (*w*/*w*)
1	6 (+1)	375 (0)	20 (+1)
2	2 (−1)	375 (0)	15 (−1)
3	4 (0)	375 (0)	17.5 (0)
4	4 (0)	250 (−1)	15 (−1)
5	4 (0)	500 (+1)	20 (+1)
6	2 (−1)	500 (+1)	17.5 (0)
7	6 (+1)	500 (+1)	17.5 (0)
8	4 (0)	375 (0)	17.5 (0)
9	2 (−1)	250 (−1)	17.5 (0)
10	6 (+1)	250 (−1)	17.5 (0)
11	6 (+1)	375 (0)	15 (−1)
12	2 (−1)	375 (0)	20 (+1)
13	4 (0)	375 (0)	17.5 (0)
14	4 (0)	250 (−1)	20 (+1)
15	4 (0)	500 (+1)	15 (−1)
(16) Pure Epoxy	0	0	0

**Table 4:** Buckling factor results using ANSYS.

Trials	Young’s Modulus (MPa)	Poisson’s Ratio	Buckling Factor
1	6848.7	0.35	87.54
2	5249.27	0.34	66.54
3	10,527.24	0.35	134.06
4	8176.21	0.35	104.51
5	8169.62	0.34	103.60
6	6239.62	0.35	79.75
7	8510.55	0.36	109.68
8	10,527.24	0.34	133.50
9	6618.06	0.35	84.59
10	8311.05	0.34	105.39
11	5505.91	0.36	70.96
12	7243.13	0.34	91.85
13	10,527.24	0.35	134.56
14	9734.19	0.34	123.44
15	7054.35	0.36	90.91
Pure Epoxy (16)	4337.98	0.35	55.44

**Table 5:** Critical Buckling load results using ANSYS.

Trials	Young’s Modulus (MPa)	Poisson’s Ratio	Critical Buckling Load
1	6848.7	0.35	875.40
2	5249.27	0.34	665.67
3	10,527.24	0.35	1345.60
4	8176.21	0.35	1045.08
5	8169.82	0.34	1036.03
6	6239.62	0.35	797.55
7	8510.55	0.36	1096.79
8	10,527.24	0.34	1335.00
9	6618.06	0.35	845.92
10	8311.05	0.34	1053.94
11	5505.91	0.36	709.57
12	7243.13	0.34	918.51
13	10,527.24	0.35	1348.60
14	9734.19	0.34	1234.40
15	7054.35	0.36	909.12

**Table 6:** Comparison of Numerical and Theoretical values of Buckling Load.

S.NO	Young’s Modulus (MPa)	Poisson’s Ratio	Theoretical Buckling Load (N)	Numerical Buckling Load (N)	Error Percentage
1	5249.27	0.34	670.45	665.67	0.71%
2	10,527.24	0.35	1340.90	1345.60	0.35%
3	8311.05	0.34	1064.83	1053.94	1.02%

**Table 7:** Comparing the Critical Buckling Load of the FEA Model and the Regression Equation.

Run Order	Sodium Hydroxide (%)	Fiber Diameter	Fiber Volume	FEA (Critical Buckling Load)	Regression Equation (Critical Buckling Load)	Percentage Deviation
1	15.0	250	4	1045.08	991.68	5.38
2	20.0	250	4	1234.40	1207.42	2.20
3	15.0	500	4	909.12	937.05	2.98
4	20.0	500	4	1036.03	1090.30	4.9
5	15.0	375	2	665.67	615.86	8.08
6	20.0	375	2	918.52	843.86	8.80
7	15.0	375	6	709.57	784.96	9.60
8	20.0	375	6	875.40	925.96	5.40
9	17.5	250	2	845.92	951.76	11.10
10	17.5	500	2	797.55	820.39	2.70
11	17.5	250	6	1053.94	1031.16	2.13
12	17.5	500	6	1096.79	991.49	10.6
13	17.5	375	4	1345.60	1342.52	0.22
14	17.5	375	4	1335.00	1342.52	0.56
15	17.5	375	4	1345.60	1342.52	0.22

**Table 8:** Model Summary.

S	R-sq.	R-sq. (adj.).
96.84	93.45%	81.67%

**Table 9:** Demonstrates Input parameters to OBT the Optimal Result.

Solution	NaoH%	Fiber Diameter (µm)	Fiber Volume%	Response (Q_e_) (N)	Composite Fit Desirability
1	18.03	333.3	4.141	1361.65	1

**Table 10:** Multiple Response Prediction.

Variable	Setting
NaoH	18.03
Fiber Diameter	333.3
Fiber Volume	4.14

**Table 11:** Optimum Response Prediction.

Response	Fit SE	95% CI	95% PI
Response Qe.	1361.6	53.6 (1224.0,1499.3)	1077.0,1646.3

**Table 12:** Comparison between the data from ANSYS and Response optimization.

S.NO	ANSYS (Critical Buckling Load) N	Optimization (Critical Buckling Load) N	Percentage Deviation (%)
1.	1351.1	1361.64	0.78

**Table 13:** Output Data.

S.NO	NaOH %	Fiber Volume %	ANSYS (Critical Buckling Load) N	Buckling Factor
1.	18.30	4.14	1351.1	135.11

## Data Availability

Not applicable.
